# Phase III Trial of PROSTVAC in Asymptomatic or Minimally Symptomatic Metastatic Castration-Resistant Prostate Cancer

**DOI:** 10.1200/JCO.18.02031

**Published:** 2019-02-28

**Authors:** James L. Gulley, Michael Borre, Nicholas J. Vogelzang, Siobhan Ng, Neeraj Agarwal, Chris C. Parker, David W. Pook, Per Rathenborg, Thomas W. Flaig, Joan Carles, Fred Saad, Neal D. Shore, Liddy Chen, Christopher R. Heery, Winald R. Gerritsen, Frank Priou, Niels C. Langkilde, Andrey Novikov, Philip W. Kantoff

**Affiliations:** ^1^ National Institutes of Health, Bethesda, MD; ^2^ Aarhus Universitetshospital, Åarhus, Denmark; ^3^ Comprehensive Cancer Centers of Nevada, Las Vegas, NV; ^4^ St John of God Subiaco Hospital, Subiaco, Western Australia, Australia; ^5^ University of Utah Huntsman Cancer Institute, Salt Lake City, UT; ^6^ Royal Marsden Hospital, Sutton, United Kingdom; ^7^ Monash Medical Centre, Bentleigh, Victoria, Australia; ^8^ Herlev Hospital, Herlev, Denmark; ^9^ University of Colorado Cancer Center, Aurora, CO; ^10^ Vall d'Hebron Institute of Oncology, Vall d'Hebron University Hospital, Barcelona, Spain; ^11^ Centre Hospitalier de l'Université de Montréal, Montréal, Quebec, Canada; ^12^ Carolina Urologic Research Center, Myrtle Beach, SC; ^13^ Bavarian Nordic, Morrisville, NC; ^14^ Radboudumc, Nijmegen, the Netherlands;; ^15^ Centre Hospitalier Départemental, La Roche sur Yon, France; ^16^ Aalborg Universitethospital, Aalborg, Denmark; ^17^ North-Western State Medical University, Saint Petersburg, Russia; ^18^ Memorial Sloan Kettering Cancer Center, New York, NY

## Abstract

**PURPOSE:**

PROSTVAC, a viral vector–based immunotherapy, prolonged median overall survival (OS) by 8.5 months versus placebo in metastatic castration-resistant prostate cancer in a phase II study. This phase III study further investigated those findings.

**PATIENTS AND METHODS:**

Patients were randomly assigned to PROSTVAC (Arm V; n = 432), PROSTVAC plus granulocyte-macrophage colony-stimulating factor (Arm VG; n = 432), or placebo (Arm P; n = 433), stratified by prostate-specific antigen (less than 50 ng/mL *v* 50 ng/mL or more) and lactate dehydrogenase (less than 200 *v* 200 U/L or more). Primary end point was OS. Secondary end points were patients alive without events (AWE)—namely, radiographic progression, pain progression, chemotherapy initiation, or death—at 6 months and safety. The study design was a superiority trial of PROSTVAC (Arm V or Arm VG) versus Arm P. Three interim analyses were planned.

**RESULTS:**

At the third interim analysis, criteria for futility were met and the trial was stopped early. Neither active treatment had an effect on median OS (Arm V, 34.4 months; hazard ratio, 1.01; 95% CI, 0.84 to 1.20; *P* = .47; Arm VG, 33.2 months; hazard ratio, 1.02; 95% CI, 0.86 to 1.22; *P* = .59; Arm P, 34.3 months). Likewise, AWE at 6 months was similar (Arm V, 29.4%; odds ratio, 0.96; 95% CI, 0.71 to 1.29; Arm VG, 28.0%; odds ratio, 0.89; 95% CI, 0.66 to 1.20; placebo, 30.3%). Adverse events were similar for the treatment and placebo groups, with the most common being injection site reactions (62% to 72%) and fatigue (21% to 24%). Arrhythmias were the most common cardiac-related events (1.4% to 3.5%). There were no reports of either myocarditis or pericarditis. Serious treatment-related events occurred in less than 1% of all patients.

**CONCLUSION:**

Whereas PROSTVAC was safe and well tolerated, it had no effect on OS or AWE in metastatic castration-resistant prostate cancer. Combination therapy is currently being explored in clinical trials.

## INTRODUCTION

The treatment algorithm for metastatic castration-resistant prostate cancer (mCRPC) continues to evolve as research brings new survival-prolonging therapies to the clinic. The mainstay of treatment for mCRPC is androgen-deprivation therapy through pharmacologic and surgical strategies.^1,2^ During the last decade, second-generation antiandrogen agents—abiraterone and enzalutamide—have received US Food and Drug Administration approval for mCRPC.^3-6^ Other treatment modalities for mCRPC that have demonstrated overall survival (OS) benefits include chemotherapy (docetaxel and cabazitaxel),^7-9^ immunotherapy (sipuleucel-T),^10^ and targeted α-therapy (radium-223).^11^

PROSTVAC is an active immunotherapy vaccine that contains prostate-specific antigen (PSA) as the tumor-associated antigen used to generate a T-cell response against prostate cancer. PROSTVAC is composed of a heterologous prime-boost regimen using two different live poxviral-based vectors: PROSTVAC-V, a recombinant vaccinia virus (rilimogene galvacirepvec), and PROSTVAC-F, a recombinant fowlpox virus (rilimogene glafolivec). Both vectors contain transgenes for human PSA and three costimulatory molecules for T cells—collectively referred to as TRICOM: B7.1, leukocyte function-associated antigen-3, and intercellular adhesion molecule-1—to enhance immune activation.^12^ On the basis of the hypothesis of potential enhancement of T-cell responses, PROSTVAC has been evaluated in clinical trials in combination with granulocyte-macrophage colony-stimulating factor (GM-CSF),^13,14^ a cytokine with immunomodulatory activity^15^; however, the necessity of GM-CSF was not established definitively in these earlier studies.

Results from a randomized, double-blind, phase II trial generated the hypothesis that PROSTVAC might prolong OS, although it did not prolong progression-free survival, the study’s primary end point, or produce any objective tumor responses.^14,16^ To validate this hypothesis and confirm the role of GM-CSF as an adjuvant, we designed a phase III trial, the results of which are reported here.

## PATIENTS AND METHODS

### Patients

Eligibility criteria included men age 18 years or older with documented asymptomatic or minimally symptomatic evidence of mCRPC and documented progressive disease (either radiologic or by PSA progression), castrate testosterone level less than 50 ng/dL, current use of a gonadotropin-releasing hormone agonist or antagonist (unless surgically castrated), and chemotherapy naïve for metastatic prostate cancer (neoadjuvant or adjuvant chemotherapy for primary prostate cancer was permitted if completed more than 3 years before screening). A 6-week washout period was required for patients on antiandrogen therapy—4 weeks if on flutamide. Concomitant medications to prevent bone loss or skeletal-related events, including bisphosphonates and denosumab, as well as palliative radiotherapy were permitted during the trial.

Exclusion criteria included cancer-related pain that required scheduled opioid narcotics—as needed two times per week or fewer was permitted—and current or prior use of sipuleucel-T for prostate cancer.

### Study Design

This was a phase III, international, multicenter, randomized, double-blind, placebo-controlled study. Patients were stratified by PSA (less than 50 ng/mL *v* 50 ng/mL or more) and lactate dehydrogenase (less than 200 U/L *v* 200 U/L or more) at screening and block randomly assigned 1:1:1 to PROSTVAC plus GM-CSF (Arm VG), PROSTVAC plus placebo GM-CSF (Arm V), or vaccine placebo plus placebo GM-CSF (Arm P) using an interactive voice response system from a randomization list generated by a third-party vendor. GM-CSF (sargramostim; Leukine [Sanofi, Bridgewater, NJ]; 250 μg, lyophilized), a glycosylated, recombinant human GM-CSF, was manufactured by Genzyme. GM-CSF placebo was USP grade or equivalent bacteriostatic sodium chloride (saline) for injection. GM-CSF was added as adjuvant therapy to one treatment arm to explore whether this immune modulator enhanced the activity of PROSTVAC, as data on its potential benefit in cancer therapy are inconsistent.^13,17-19^ An empty vector fowlpox was used for the placebo vaccination as it is nonreplicating in humans; has minimal safety risks, including no known effects on cancer progression; and an adverse effect profile, including injection site reactions, that is overlapping and generally indistinguishable from subcutaneously administered vaccinia. This trial is registered with the European Clinical Trial Database (EudraCT 2010-021196-85).

The study consisted of three periods: screening, treatment, and long-term follow-up (LTFU). During the treatment phase, a total of seven vaccinations were subcutaneously administered—a single priming immunization of PROSTVAC-V or placebo in week 1 followed by six boosting immunizations with PROSTVAC-F or placebo administered in weeks 3, 5, 9, 13, 17, and 21. Subcutaneous GM-CSF or placebo was administered—within 5 mm of the original injection site—on the day of immunization and for 3 consecutive days thereafter beginning with week 1, for a total of 28 doses. After the end-of-treatment visit—week 25/early termination—all patients were automatically entered into the LTFU phase, with study visits occurring every 6 months. During the treatment phase (approximately 5 months), chemotherapy, immunotherapy (eg, sipuleucel-T) or immunosuppressive therapy (eg, etanercept or natalizumab), systemic corticosteroids (daily or continued use every other day for more than 14 days), anticancer radionuclides, and secondary anticancer hormonal treatments (eg, abiraterone) were prohibited. During LTFU, patients received standard-of-care treatment as determined by the investigator.

The study was approved by local or central institutional review boards or ethics committees for each participating site and conducted according to the provisions of the Declaration of Helsinki and Good Clinical Practice Guidelines of the International Conference on Harmonization. All patients provided written informed consent before any screening procedures were initiated.

### Outcomes

Primary end point of the study was OS, defined as the time between the date of random assignment and the date of death as a result of any cause. Secondary end point was the proportion of patients alive without events (AWE) —namely, radiographic progression, pain progression, initiation of chemotherapy for prostate cancer, or death—at 6 months post–random assignment. Safety end points included adverse events, vital signs, and 12-lead ECG. Adverse events were graded according to the National Cancer Institute Common Terminology Criteria for Adverse Events (version 4.0). Tumor response was according to Response Evaluation Criteria in Solid Tumors (RECIST) criteria 1.1. Exploratory/other end points included survival on the basis of HLA-A2 status, postvaccination cancer treatments (abiraterone acetate, enzalutamide, or sipuleucel-T), and growth rate constant calculated using PSA values from screening through week 25/end-of-treatment visit.

### Statistical Analysis

All efficacy analyses were conducted using the intention-to-treat population, defined as all randomly assigned patients, with analysis according to the randomized treatment arm. Safety analyses were conducted using the full analysis set population, defined as all patients who initiated treatment. Primary analysis was based on a stratified log-rank test. Hazard ratio was estimated using stratified Cox proportional hazards regression analysis, with ties handled by the exact method. Survival data were plotted using the Kaplan-Meier method. An estimated 534 deaths in a between-arm comparison was needed to achieve a target hazard ratio of 0.68 with a one-sided type I error of 0.0125 and a target power of 85% or greater. The number of patients per arm was set at 400 for an approximate total of 1,200 patients.

Two main overall comparisons of the primary end point were planned—one between Arm V and Arm P and the other between Arm VG and Arm P—and these were performed using a Bonferroni correction for the overall type I error probability such that the probability for each one-sided comparison would not exceed 0.0125 (0.025/2). Trial success was defined as meeting the statistical criterion for either comparison. The same comparisons performed for survival were performed for AWE using the same significance levels. AWE end point was analyzed using a logistic regression model stratified by randomization strata. The 95% CI for the odds ratio (OR) estimate was computed as the measure of effect size.

Three interim analyses were planned for OS superiority and futility after 321, 481, and 641 deaths, which represented 40%, 60%, and 80% of the deaths required for final analysis (801 deaths in the three arms). In addition to the O’Brien-Fleming efficacy stopping boundaries to control the overall type I error, a significance level of 0.00001 was used for detection of futility at each interim analysis. An unblinded independent third-party vendor performed interim analyses. Results were assessed by an independent data monitoring committee. SAS (SAS/STAT User’s Guide, Version 9.2; SAS Institute, Cary, NC) was used for all statistical analyses.

## RESULTS

### Patient Characteristics and Disposition

**FIG 1.**
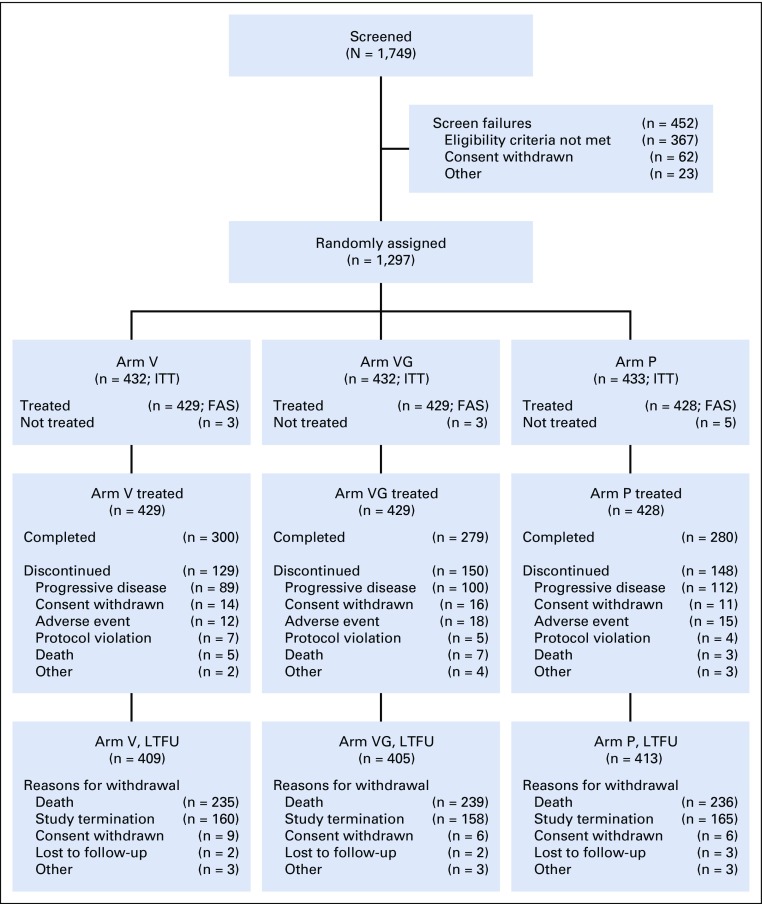
Patient disposition. Safety was assessed using the full analysis set population (FAS). Arm P, placebo; Arm V, PROSTVAC; Arm VG, PROSTVAC + granulocyte-macrophage colony-stimulating factor; ITT, intention-to-treat population, LTFU, long-term follow-up.

A total of 1,749 patients from 105 sites in 16 countries were screened, and 1,297 were randomly assigned to one of the three treatment arms, comprising the intention-to-treat population. Of these, 11 patients were never treated; therefore, the full analysis set/safety population consisted of 1,286 patients. The first patient was screened in November 2011 and the last patient completed the treatment phase in July 2015. After the third interim analysis, criteria for futility were met and, on the recommendation of the DMC, the trial was stopped early (September 25, 2017) and the date of last follow-up was October 02, 2017. A flowchart of patient enrollment and disposition is shown in Figure 1. A similar proportion of treated patients in all arms (65% to 70%) completed the treatment period. Reasons for discontinuation were also similar among the study arms, with progressive disease (23.4%; 301 of 1,286 patients) being the most common, whereas adverse events accounted for 3.5% (45 of 1,286 patients) of all treatment discontinuations.

The majority of treated patients in each arm (94% to 96%) entered the LTFU phase (Fig 1). As follow-up was to continue until death as a result of any cause, death was the most common reason for discontinuation from LTFU. Given the DMC recommendation to stop the trial early, the second most common reason was study termination by sponsor.

Patients were evenly distributed across the treatment arms by demographic and disease characteristics (Table 1). Mean age of the study population was 71 years (range, 45 to 93 years), and the primary site of metastasis in all study arms was bone, occurring in approximately 75% of patients. Approximately one third of all patients reported a history of cardiac disorders, with coronary artery disorders being the most common at approximately 20%.

**TABLE 1.**
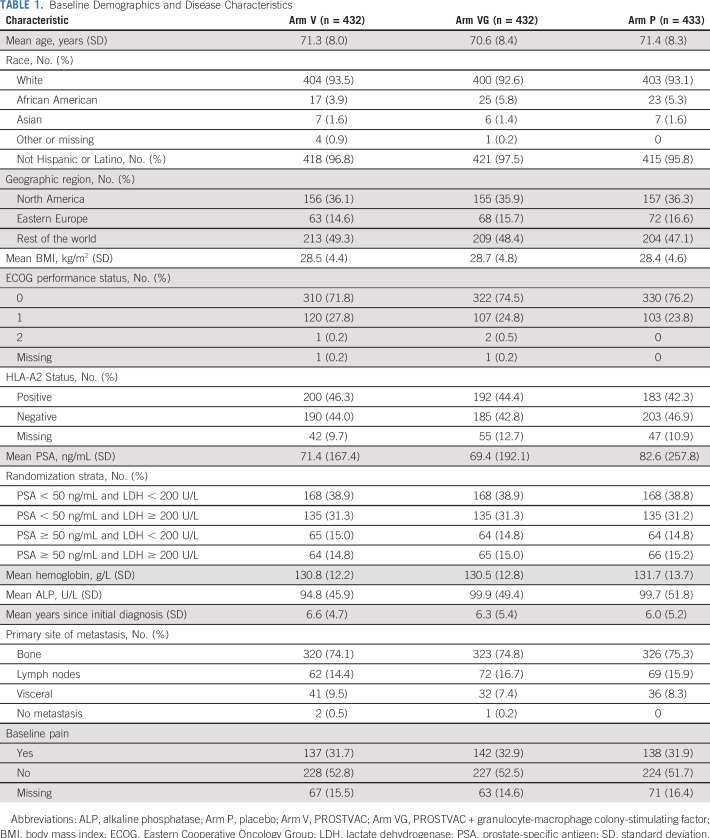
Baseline Demographics and Disease Characteristics

### Primary End Point

**FIG 2.**
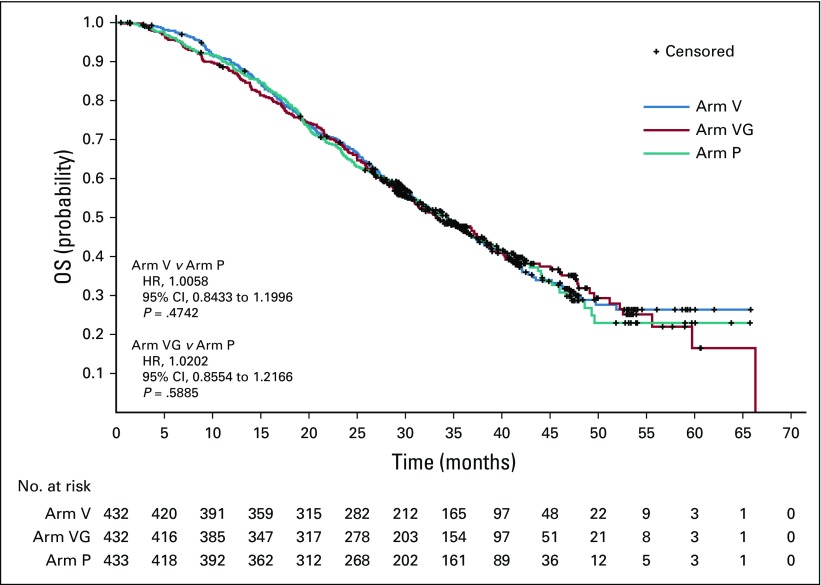
Kaplan-Meier estimates for overall survival (OS). Arm P, placebo; Arm V, PROSTVAC; Arm VG, PROSTVAC + granulocyte-macrophage colony-stimulating factor; HR, hazard ratio.

As shown in the Kaplan-Meier curves (Fig 2), the two active arms were not different from placebo with respect to OS. Median OS was 34.4 months in Arm V, 33.2 months in Arm VG, and 34.3 months in Arm P. The hazard ratio for comparison with placebo was 1.01 (95% CI, 0.84 to 1.20; *P* = .47) for Arm V and 1.02 (95% CI, 0.86 to 1.22; *P* = .59) for Arm VG.

### Secondary End Point

Likewise, the proportion of patients AWE at 6 months postrandomization was similar across treatment arms (Arm V; 29.4%; OR, 0.96; 95% CI, 0.71 to 1.29; Arm VG: 28.0%; OR, 0.89; 95% CI, 0.66 to 1.20; Arm P: 30.3%; Table 2). The most common event was radiographic progression, which occurred in approximately 60% of patients, followed by pain progression, which ranged from 6.5% in Arm V to 10.4% in Arm P.

**TABLE 2.**
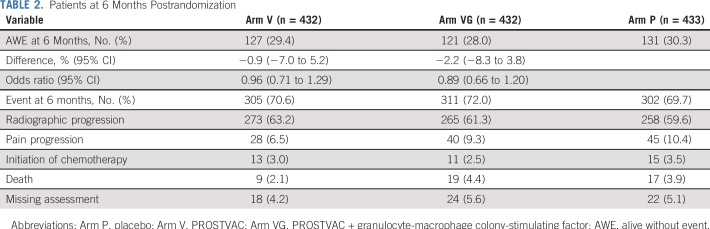
Patients at 6 Months Postrandomization

### Exploratory/Other End Points

HLA-A2 status—positive versus negative—did not influence OS (Table 3). Whereas there were significant differences in OS between postvaccination cancer therapy—yes versus no—and between tumor growth rate quartiles, there were no differences between treatments within each subgroup (Table 3, Data Supplement Figures A2, A3).

**TABLE 3.**
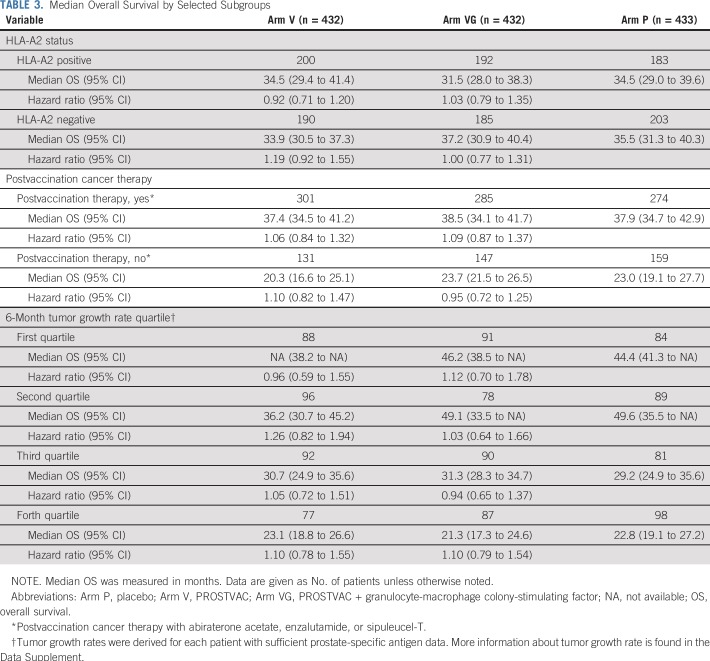
Median Overall Survival by Selected Subgroups

Tumor response results were similar across treatments (Table 4, Data Supplement Figure A1). At week 25/end of treatment, only one patient achieved a complete response and this was observed in a placebo-treated patient. A best response of partial response was observed in less than 1% of patients in each study arm.

**TABLE 4.**
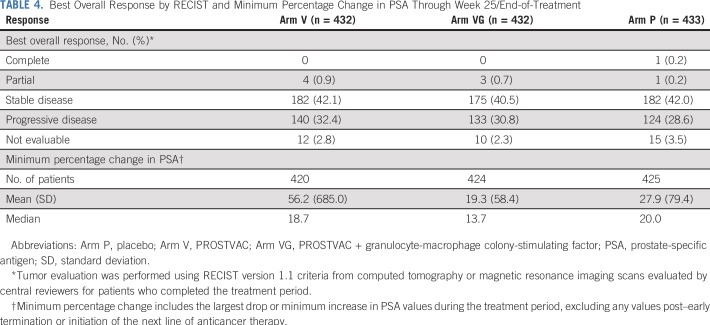
Best Overall Response by RECIST and Minimum Percentage Change in PSA Through Week 25/End-of-Treatment

### Safety

Nearly all treated patients—approximately 91%—experienced at least one treatment-emergent adverse event (TEAE). As expected, the most common events in all three treatment arms were injection site reactions, which occurred in 62% to 72% of patients. The most common noninjection site event for all treatment arms was fatigue, which occurred in 21% to 24% of patients. Whereas at least one grade 3 or greater TEAE was reported in 21% to 23% of patients, the majority (more than 75%) of all TEAEs were mild in severity (grade 1). Adverse events that led to treatment discontinuation occurred in 4.9% (21 of 429 patients), 6.5% (28 of 429 patients), and 5.8% (25 of 428 patients) of patients in Arm V, Arm VG, and Arm P, respectively. In only a minority of patients were the events considered treatment related (Arm V: 0.9% [four of 429 patients]; Arm VG: 3.0% [13 of 429 patients]; Arm P: 1.6% [seven of 428 patients]). The most common TEAEs that led to treatment discontinuation in the study arms were bone pain (n = 6; two patients in each treatment arm) and spinal cord compression (n = 6; one patient in each PROSTVAC arm and four patients in the placebo arm). A total of nine patients (Arm V, n = 3; Arm VG, n = 4; Arm P, n = 2) experienced 10 serious adverse events that were considered by study investigators to be treatment related, but no single event occurred in more than one patient each per study arm. The most common treatment-related TEAEs by toxicity grade are shown in Table 5. Overall, treatment-related adverse events were more commonly observed in Arm VG (79.3%) compared with Arm V (72.7%) or placebo (70.6%), and this was especially noticeable with pyrexia (19.3%, 6.5%, and 11.0%, respectively) and injection site reactions. Cardiac disorders occurred in less than 5% of all treated patients, with no differences across treatment arms. Arrhythmias were the most commonly reported cardiac-related TEAEs, occurring in 1.4% (Arm VG) to 3.5% (placebo) of all patients. There were no reports of myocarditis or pericarditis.

**TABLE 5.**
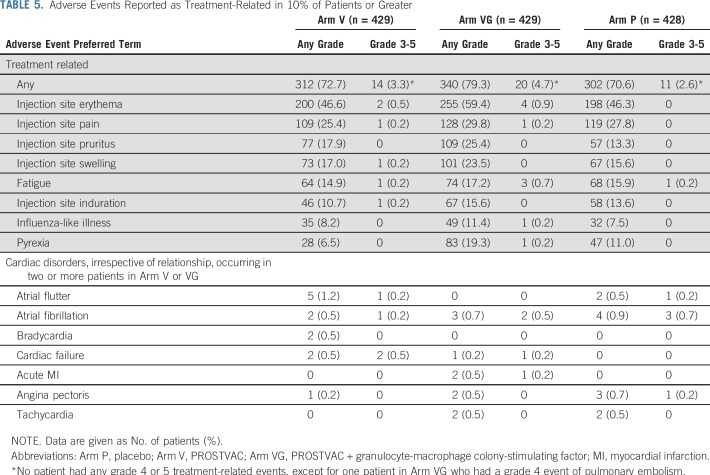
Adverse Events Reported as Treatment-Related in 10% of Patients or Greater

## DISCUSSION

In this phase III study, PROSTVAC did not meet the primary OS end point compared with control in patients with asymptomatic or minimally symptomatic mCRPC. These results did not support the positive signal from the randomized phase II study.^14^

A few possibilities may account for these findings: the phase II finding generated a false-positive signal as a result of being underpowered for an OS comparison, the relatively small sample size (82 PROSTVAC-treated *v* 40 placebo-treated patients), and/or potential observer bias (long-term OS data were collected after database lock and study unblinding).^14,16^ Moreover, an imbalance in prognostic factors, both known and unknown, may have negatively affected the observed median OS in the phase II control arm (16.6 months). This was lower than expected compared with the Halabi prognostic nomogram-predicted OS (PSA, lactate dehydrogenase, alkaline phosphatase, and hemoglobin; 20.4 months).^14,20^ Survival in the control arm relative to expected outcomes may be the origin of the flawed comparison, which is supported by the OS in the control arm of the sipuleucel-T pivotal trial (21.7 months).^10^ Finally, docetaxel was the only life-prolonging treatment available during our phase II study. From the time the phase III protocol was finalized (2010) until the last patient was randomly assigned (January 2015), multiple treatments became available—cabazitaxel,^9^ sipuleucel-T,^10^ abiraterone acetate^3,4^ enzalutamide,^5,6^ and radium-223^11^—and we observed an approximate 3-year median OS in the placebo group. It should be noted that in similar predocetaxel patient populations, OS benefit in clearly clinically active agents—abiraterone and enzalutamide—was less robust than in postchemotherapy, likely because of subsequent therapies.^4,6^ It is possible that these life-prolonging therapies negatively affected the likelihood of this trial achieving positive results. Despite this statistical possibility, the lack of any signal of efficacy indicates that PROSTVAC, as a single agent in this setting, seems to be ineffective at a level that would justify the treatment of unselected patients. Preclinical evidence suggested that PROSTVAC generated fully functional T cells that were capable of trafficking to and infiltrating into murine models of prostate cancer.^21^ Accumulated immune data from multiple National Cancer Institute clinical trials also suggested that antitumor immune responses could be identified in the peripheral blood.^22^ The observed lack of clinical signal suggests that either the immune responses generated in this study were not sufficient or there were other negative regulatory influences in the tumor microenvironment that prevented clinically relevant immune-mediated killing.

GM-CSF has been adopted by cancer vaccine developers on the basis of its properties of differentiation, migration, and activation of dendritic cells, including an enhancement of antigen cross-presentation^23,24^; however, biologic effects of GM-CSF also stimulate other myeloid cells, including myeloid-derived suppressor cells, which inhibit the functionality of T cells.^25-27^ We observed an increase in adverse events in the treatment arm that contained GM-CSF, and as a result of the overall lack of efficacy of PROSTVAC versus placebo, our data do not support any definitive recommendation about GM-CSF, positive or negative. Therefore, use of GM-CSF as a vaccine adjuvant must still be considered investigational.

A correlation between growth rate constant and survival has been reported in a retrospective analysis of five phase II clinical trials.^28^ Our data are in agreement, showing a strong correlation between slower growth rate constant and longer survival regardless of treatment group. Our data, however, cannot confirm the hypothesis that patients with more indolent mCRPC derive greater benefit from vaccine therapy than do those with poorer prognostic factors,^29^ considering the absence of an OS benefit in the entire study population. Nonetheless, we have demonstrated that tumor growth rate, as measured by the model used in this study, is a reasonable predictor of the risk of death on a population level. This tool could be used in clinical trials to stratify or select patients who are at high or low risk for death, as appropriate for the primary study end point.

PROSTVAC was found to be safe and well tolerated, with no unexpected TEAEs, which confirmed the result of earlier studies.^13,14,30^ Overall, TEAEs were similar to placebo and most commonly related to injection site reactions. An increased risk for myocarditis or pericarditis has been observed with replicating vaccinia virus strains used as preventive vaccines for smallpox (variola).^31-33^ As PROSTVAC uses a replicating vaccinia strain as the vector, cardiac adverse events are a safety issue of special interest. No signal of cardiotoxicity was observed in our study, even with the population being in the high-risk category for cardiovascular events—that is, male, older than age 65 years (approximately 75% of our population were older than age 65 years).^34^

Therapeutic cancer vaccines remain a valid immunotherapy option for prostate cancer, as supported by the survival benefit of sipuleucel-T.^10^ The choice of proper target antigens and adjuvant components that can overcome immune resistance within the tumor microenvironment are of critical importance. Along with the confounding factors that surround additional treatment options that became available during our study, it is not known, for instance, if the selection of prostatic acid phosphatase in sipuleucel-T versus PSA in PROSTVAC played a role in efficacy evaluation.

In conclusion, we observed that vaccines induce T cells that are capable of infiltrating tumors, but that this immune response does not translate into clinical benefit poses a major challenge to the immunotherapy community. Historical data have shown that PROSTVAC is capable of generating specific T-cell responses against PSA as well as cascade antigens,^22^ indicating that the poxvirus platform has the potential to induce clinical benefit in the right context—that is, with different antigen targets, in other disease settings, and in combination with checkpoint inhibition. This possibility is being evaluated in ongoing clinical trials.
